# Efficient Cumulant-Based Automatic Modulation Classification Using Machine Learning

**DOI:** 10.3390/s24020701

**Published:** 2024-01-22

**Authors:** Ben Dgani, Israel Cohen

**Affiliations:** Andrew and Erna Viterbi Faculty of Electrical & Computer Engineering, Technion-Israel Institute of Technology, Haifa 3200003, Israel

**Keywords:** cumulants, modulation classification, machine learning

## Abstract

This paper introduces a new technique for automatic modulation classification (AMC) in Cognitive Radio (CR) networks. The method employs a straightforward classifier that utilizes high-order cumulant for training. It focuses on the statistical behavior of both analog modulation and digital schemes, which have received limited attention in previous works. The simulation results show that the proposed method performs well with different signal-to-noise ratios (SNRs) and channel conditions. The classifier’s performance is superior to that of complex deep learning methods, making it suitable for deployment in CR networks’ end units, especially in military and emergency service applications. The proposed method offers a cost-effective and high-quality solution for AMC that meets the strict demands of these critical applications.

## 1. Introduction

The use of smart transmitters, adaptive receivers and intelligent communication networks has revolutionized radio communication in the modern era. These technologies have helped us optimize our usage of wireless channels. One crucial task in this regard is the implementation of intelligent radio modulation detection. Automatic modulation classification plays a vital role in various civilian and military applications, which are diverse and often massive. Established radio networks have been in operation for years and require fast and adaptive radio networks that can be used to combat various tasks daily and in times of need. To achieve this, the SDR platform is considered a feasible option.

Software-defined radio (SDR) is a communication platform that works through software programs rather than traditional hardware components. This means that the radio’s functions can be adapted and changed dynamically based on different networks and tasks. SDR is considered the modern realization of the term “software radio”, which was coined by Mitola [1]. By converting signal processing to the digital domain, SDR-based networks can incorporate complex algorithms that improve the communication rate, accuracy, and distance.

One example of the benefits of SDR is the concept of Cognitive Radio, which was also introduced by Mitola in 1999 [2]. The idea behind Cognitive Radio is to develop a radio unit that can manage its communication network dynamically and automatically using software programs. The reason for this is to solve the problem of spectral congestion in modern communication. As the number of daily users increases, spectrum resources become more expensive and less available. By using Cognitive Radio, the radio unit can adapt to its environment and establish unlicensed communication networks that share the same wireless spectrum with other users. This helps solve the problem of spectral congestion and makes it easier for unlicensed users, such as the military and emergency services, to communicate effectively in times of crisis.

Setting up the shared network involves a method called spectrum sensing, where the Cognitive Radio unit can detect and analyze the available spectrum, and then manage the communication network accordingly. By utilizing software instead of expensive and complex hardware, Cognitive Radio offers a more efficient and cost-effective solution to the problem of spectral congestion. During the spectrum sensing step, a Cognitive Radio performs a scan of all available channels to find “empty holes”, or unused frequencies in the spectrum. By sensing the channel parameters, the adaptive qualities of the Cognitive Radio enable it to dynamically change the modulation scheme used for transmission in order to improve the transmission rates and quality. However, dynamic modulations require a recognition method for the received signal modulation scheme, which is known as automatic modulation classification, or modulation recognition and modulation detection in the literature [3,4,5]. Spectrum sensing is essential for adaptive networks that must change according to their location without causing any interruption to local users’ daily communication needs, especially in times of emergency, where every message counts.

Automatic modulation classification (AMC) is a process used to detect and identify the unknown modulation scheme of a radio signal. It has been around since the 1980s [6,7] but gained more popularity after the publication of the Cognitive Radio idea. Today, the modern AMC literature is mainly focused on deep learning and neural networks [8,9,10]. However, the classic AMC algorithms are split between two methods [11]. The process of identifying radio signals can be challenging due to the high computational complexity that certain algorithms require. The first set of methods, called likelihood-based algorithms [12], are accurate but can be expensive to implement, which is a drawback for government-funded services that rely on cost-efficient projects.

On the other hand, feature-based (FB) methods offer a more efficient approach to signal analysis. By extracting features like high-order statistical moments (HOS) or instantaneous frequency and phase parameters, these algorithms can help understand signal characteristics. FB algorithms rely on a predetermined set of features, reducing the computational burden when compared to more complex machine learning techniques. To use FB algorithms, the process starts by extracting classification features from the signal. Once the algorithm has identified the key features, it can train the selected classification algorithm. Some examples of feature-based algorithms are support vector machines (SVMs), decision trees, and K-means. After the model is trained, it can be applied with minimal complexity and runtime, making it a useful tool for identifying radio signals.

In recent years, the field of neural networks has experienced massive growth, leading to most new studies on AMC utilizing neural networks for feature extraction, classification, or both. Neural networks have demonstrated optimal results concerning classification accuracy, achieving high detection probability, even at low SNR values. However, like likelihood-based methods, neural networks can suffer from high computational complexity and require significant system storage if a deep network model is used. This can become a challenge when dealing with a quick-setup radio network that relies on end units and needs to be power- and time-efficient. This is particularly important when considering emergency services, where there is a need for a new, easy-to-set-up network that can quickly adapt itself to the spectral environment. Therefore, the training process for the desired modulation must be efficient.

This paper focuses on the FB method for the AMC, where we utilize high-order cumulants (HOCs) as key features. Cumulants are statistical measures that are alternatives to moments such as mean and variance. The HOCs are popular in the AMC literature as a detection feature [13,14], due to their additive property that separates the desired signal from its additive noise. While most of the literature discusses digital modulation classification [15,16], our work aims to include analog modulations as well. In our study, we analyze the statistics of eleven modulation schemes, explore the best classification feature for each modulation, and discover the statistical connection between pairs of modulations.

The rest of this paper is organized as follows. Section 2 presents the modulated signal models and the theory behind HOS and cumulants. In Section 3, we evaluate the simulation results for our proposed FB method. Finally, in Section 4, we discuss the conclusions from the results.

## 2. Background and Algorithm

This section is divided into four subsections to provide a comprehensive explanation of our work. The first part briefly reviews the theory of high-order statistics and the cumulants, which provide an alternative approach to the more familiar moments. In the second part, we introduce the dataset that we used for our work. The third subsection explains the theoretical aspect of the classification process. Finally, the last subsection briefly explains the method we used for our work.

### 2.1. High-Order Statistics

In statistics, moments of a random variable provide quantitative measures related to the shape of its probability density graph and the distribution behavior. Specifically, the *n*th-order moment of a random variable *x* is defined by

(1) $$m_{n}\left(x\right)=\int x^{n}f\left(x\right)dx,$$

where f(x) is the probability density function of *x*. If the *n*th-order moment integral diverges, the *n*th-order moment of the variable does not exist. If the *n*th-order moment of *x* exists, so does the (n−1)th-order moment, and thus all lower-order moments. We define a central moment by $m_{n}\left(x-\mu \right)$, which is the mean value of *x*, and define the normalized moment as $m_{n}\left(x/\sigma \right)$, where σ is the standard deviation.

The cumulants $c_{n}$ of a probability distribution are an alternative to the moments of the distribution. Any pair of probability distributions with identical moments will also share identical cumulants, and vice versa. The first, second, and third cumulants are equal to the first three central moments—known as the mean, variance, and skewness—but fourth- and higher-order cumulants diverge from the central moments. The cumulant of a random variable *x* is defined using the cumulant-generating function

(2) $$C_{t}\left(x\right)=log\left(E\left[e^{tx}\right]\right),$$

and the cumulants $c_{n}$ are obtained from a power series expansion of the cumulant-generating function:

(3) $$C_{t}\left(x\right)=\sum c_{n}\frac{t^{n}}{n!}.$$

This expansion is a Maclaurin series expansion of the logarithm function, so we obtain that the *n*th-order cumulant can be obtained by differentiating the expansion *n* times at *t* = 0. The use of cumulants instead of moments as features for AMC in the literature is usually based on their cumulative property: assume *X* and *Y* are independent random variables, then

(4) $$c_{n}\left(X+Y\right)=c_{n}\left(X\right)+c_{n}\left(Y\right).$$

Despite its benefits, the cumulant-generating function can be challenging to calculate, especially when we are not familiar with the exact distribution of the signal. However, since moments and cumulants are shared when the distribution is the same, we can use the moments to calculate the cumulants based on the work in [14,17]. We use

(5) $$\begin{array}{ccc} c_{4.0} & = & m_{4.0}-3m_{2.0}^{2} \end{array}$$

(6) $$\begin{array}{ccc} c_{4.1} & = & m_{4.1}-3m_{2.1}m_{2.0} \end{array}$$

(7) $$\begin{array}{ccc} c_{4.2} & = & m_{4.2}-{\left|m_{2.0}\right|}^{2}-2m_{2.1}^{2} \end{array}$$

(8) $$\begin{array}{ccc} c_{6.0} & = & m_{6.0}-15m_{4.0}m_{2.0}+30m_{2.0}^{3} \end{array}$$

(9) $$\begin{array}{ccc} c_{6.3} & = & m_{6.3}-9c_{4.2}m_{2.1}-6m_{2.1}^{2} \end{array}$$

(10) $$\begin{array}{ccc} c_{8.0} & = & m_{8.0}-28m_{6.0}m_{2.0}-35m_{4.2}^{2}+420m_{4.0}m_{2.0}^{2}-630m_{2.0}^{4} \end{array}$$

where $m_{q,p}$ is the mixed moment of the signal, defined as $E(x^{q}\times {\bar{x}}^{p-q})$. This set of equations enables simple and fast calculation for the HOC, which will act as features for the classification algorithm. Since our work is based on digital signals, we will use the discrete approximation for the mixed moment of N samples from a signal:

(11) $$m_{q,p}=\frac{1}{N}\sum_{n=1}^{N}x^{q}\left[n\right]\times {\bar{x}}^{p-q}\left[n\right].$$

The classification method in our paper uses the cumulants above as input features, along with the matching cumulants $C_{p,q}\left(x_{d}\right)$ for the derivative of the signal

(12) $$x_{d}\left[n\right]=x\left[n+1\right]-x\left[n\right].$$

After experimenting with different features, we added the cumulants of the derivative to the features list, including separated cumulants for the real and imaginary parts of the signal and the cumulants of the amplitude and phase of each signal. Only the derivative’s cumulants noticeably improved the classification accuracy.

When discussing AWGN (additive white Gaussian noise), the primary benefit of utilizing cumulants becomes evident. The noise is a random process that follows a normal distribution. Therefore, we can use its cumulant-generating function, which is calculated in [18,19] using

(13) $$C_{t}\left(w\right)=log\left(M_{t}\left(w\right)\right)=\mu t+\sigma^{2}\frac{t^{2}}{2},$$

where μ and σ are the mean and standard deviation, respectively. The function in (13) is a second-order polynomial, so its 3rd- and higher-order derivatives are zero-valued, meaning the HOC cumulants of AWGN are equal to zero. Hence, the HOC of the sum between AWGN and another independent random process will equal the HOC of the second random process alone. Since the derivative of AWGN is distributed the same, the derivative’s cumulants are still effective for our method under the above assumptions.

### 2.2. Modulation and Signal Model

The signals used in this paper are taken from the RadioML2018.01A dataset [20]. This dataset contains 2,555,904 frames. Each frame is 1024 IQ samples from a modulated signal, with various modulation schemes and SNR values from −20 to 30 dB. The dataset contains 24 different modulations generated using SDR under the following channel specifications:

- Selective multipath Rician fading;
- Carrier frequency offset;
- Symbol rate offset;
- Non-impulsive delay spread;
- Doppler shift;
- AWGN.

We only consider 11 modulations as the “Normal” set. This includes the following: OOK, 4ASK, BPSK, QPSK, 8PSK, 16QAM, AM-SSB-SC, AM-DSB-SC, FM, GMSK, OQPSK (We will refer to AM-SSB-SC and AM-DSB-SC as SSB and DSB, respectively). We decided to evaluate our work on the “Normal” dataset since it is considered the benchmark for classification models in the literature, in contrast to the “hard” set, which contains all 24 schemes.

### 2.3. Decision Tree

A decision tree is a flowchart-like model for supervised learning in which each internal node represents a decision test on a single feature, each branch represents the outcome of the test, and each leaf node represents a class label. The paths from root to leaf represent the classification rules. Decision trees have several advantages as a classification model, and we are focusing on two of them in this paper. The first is that training the tree model does not require a large dataset; this is a significant advantage over neural networks that are widely popular in the literature despite the need for massive data to yield results. The second advantage is the model’s simplicity, which allows it to combine with other decision techniques. The benefit of combining techniques comes when we make a tree model, where instead of a simple decision rule at each node, we can use a more complex model.

The decision tree model we used in our work is from the Python scikit-learn (Sklearn) library, which is trained based on Gini impurity. Gini impurity is a measurement for the probability of classifying the labels incorrectly. Let *A* be a random variable that takes one of *k* labels $(a_{1},a_{2},\ldots \;a_{k}$); then, we define the Gini impurity of a classification model as

(14) $$G\left(A\right)=1-\sum_{i=1}^{k}{\left(P\left(a_{i}\right)\right)}^{2}\;.$$

The training process of a decision tree contains three steps:

1. Decide the feature to split the data: for each feature, the Gini impurity is calculated, and the one for which it is minimized is selected.
2. Continue splitting the data based on 1.
3. Stop splitting the data if we reach a certain tree depth.

The decision tree model’s parameters are the comparison threshold at each node, barring the leaves, meaning the total number of parameters in a tree of depth *d* can be calculated with

(15) $$P_{n}=2^{H}-1,$$

which is also the number of calculations a single tree applies when classifying since there is no need for multiplications or additions.

### 2.4. Method

Our classification algorithm is designed for integration within SDR systems operating in Cognitive Radio networks. The workflow initiates with signal acquisition by the SDR, followed by the segmentation of acquired signals into discrete packets, typically standardized at a length of 1024 samples—although this length is variable, subject to network requirements and design. These packets serve as inputs to our classification algorithm. For each packet received, we use the set of Equations (5) through (10) to extract the features vector of the cumulants. This feature extraction process is computationally straightforward, involving a singular pass through the packet data, which aligns with the predetermined packet length of the communication network’s settings.

Subsequently, these extracted features undergo the classification phase, where the decision tree model is employed. The output of this classification stage yields the identification of one among the 11 available modulation schemes within the “Normal” modulations set. The detected modulation holds significant utility within the Cognitive Radio context, facilitating dynamic functionalities, such as adaptive frequency channel selection and network usage detection. Furthermore, the system’s training procedure can be executed proactively in controlled environments or dynamically during operational use, enabling real-time adaptation to the evolving network landscape. This adaptability ensures the system is efficient and adaptive within varying operational scenarios, allowing seamless integration into dynamic spectrum environments.

## 3. Simulation and Results

The suggested classification method requires hand-picked features for each step, so we start the simulation with a small-scale experiment. We start with an experiment to determine which of the features is optimal for the classification and detection of every single modulation from our desired set of 11 schemes. This simulation includes a simple decision tree model, where only one feature from the cumulants and derivative cumulants is used each run, where a run is defined as training and testing. Table 1 shows the top two features for each modulation scheme in terms of the detection accuracy using the simple decision tree model.

Table 1 shows the average outcomes obtained when classifying signals specifically at an SNR of 10 dB. This SNR value is deemed sufficient for a receiver to effectively detect and demodulate signals with accuracy. Utilizing the insights derived from this table, we proceed to construct the ultimate feature vector, which forms the foundational input for our classification algorithm.

This feature vector is meticulously composed of a consolidated set of cumulants, as illustrated in the table, representing a combined and optimized selection based on their performance at the specified SNR of 10 dB. This selection process is pivotal in crafting an efficient and discriminative feature set, chosen explicitly for its effectiveness in signal classification within this specific SNR context. By amalgamating these cumulants into a unified feature vector, we aim to encapsulate the most informative and distinguishing signal characteristics essential for accurate modulation classification at this SNR threshold.

**Figure 1:**
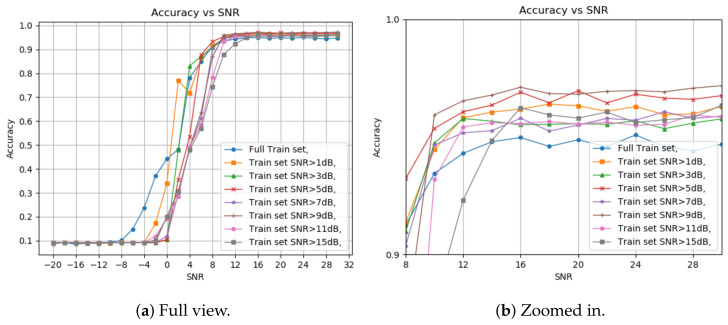
$P_{d}$ based on train set SNR threshold. (**a**) Full view; (**b**) zoomed-in view.

The next part of the simulation is the picking process of the training set for the model. It is widely agreed that for training machine learning models, the algorithm randomly picks the training set, out of the total database. However, in our work, we consider training our model with only a subset of the data. This subset includes all the sampled signals with SNR values higher than a certain threshold. The reasoning behind our decision comes from the fact that when we study different modulation schemes, we can see that each scheme has a minimal SNR requirement for adequate demodulation, which means that using signals with an SNR below that threshold might affect our model training in negative ways. Based on these facts, we split the data into two separate groups: high SNR signals and low SNR signals. In order to pick an SNR threshold for the splitting, we conducted our second experiment. Figure 1 shows the classification results when we train the model using a signal with SNR higher than varying thresholds; we also include a zoomed-in view of the section where the classification accuracy is the highest.

First, picking a train set from all the SNR values gives better results at a negative SNR but deteriorates when reaching a positive value as expected. Another conclusion is that using over 5 dB as the threshold gives lesser accuracy for high and low SNR test samples. The final choice of threshold was between 1 dB and 5 dB. Although the 1 dB threshold yields better accuracy at lower SNR levels, a slight improvement in the detection accuracy is observed starting from 4 dB and higher with the 5 dB threshold. This result supports the assumption that modulation schemes have minimal SNR requirements for perfect demodulation. The depth of the decision tree was not considered for the first test. So, we want to pick a balanced tree, deep enough to view all the features we chose before but not too deep, which can result in overfitting and high complexity.

**Figure 2:**
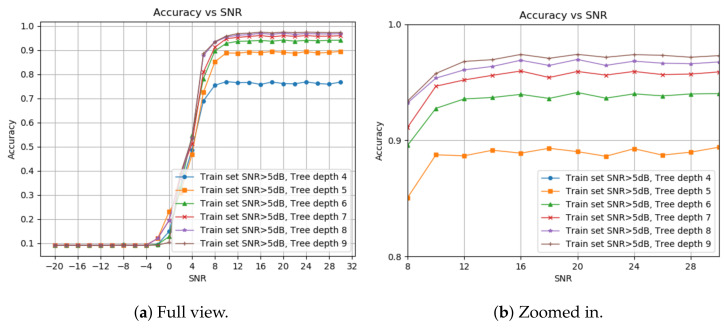
$P_{d}$ based on tree depth. (**a**) Full view; (**b**) zoomed-in view.

Figure 2 shows the detection probability under different decision tree depths. We start at four layers since this is the minimal depth for 11 labels, and stop at nine, as we see no significant improvement from eight. The optimal depth for each application can be chosen based on the memory and runtime requirements. We pick an 8-deep tree with a higher than 5 dB SNR train set.

**Figure 3:**
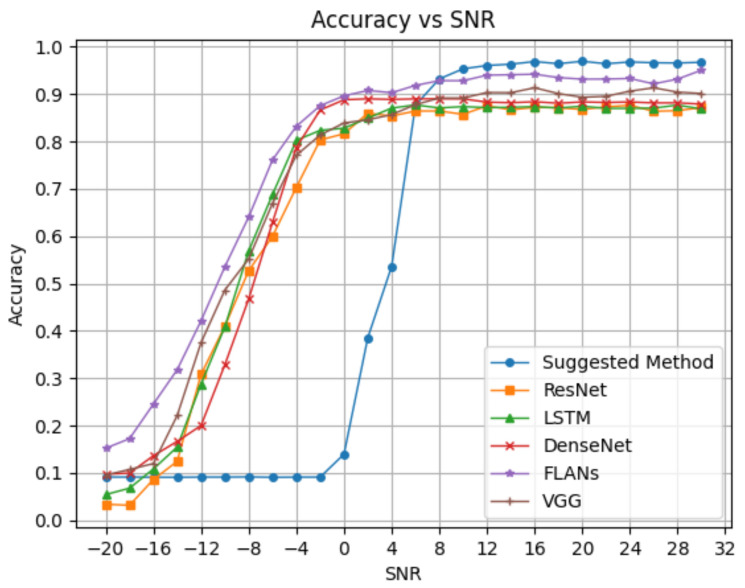
$P_{d}$ comparison for the suggested method.

We aim to compare our results to state-of-the-art deep learning models. First, we compare our method with three different state-of-the-art models of deep neural networks as examples. The models we examine are ResNet [21], DenseNet [22], LSTM [10], FLANs [8] and VGG [23]. We aim to compare our model to the neural networks in two manners. Figure 3 shows the first comparison of neural networks in the classification accuracy.

We can see the advantage of deep neural networks in the negative SNR zone, while our method fails in comparison. However, we already discussed why classification at negative SNR is usually irrelevant in terms of perfect demodulation. Starting from a splitting threshold of 6 dB SNR, our suggested method yields better detection accuracy than the popular NN methods by a margin of almost 8%.

The secondary comparison with the neural network models primarily centers on the parameters or memory requirements. We use Equation (15) to compute the total parameters necessary for diverse tree depths, subsequently contrasting these values in Table 2 alongside the proposed NN models.

Our model, even with the inclusion of additional layers, consistently maintains a parameter count ranging in the range of a few hundreds. In contrast, the NN models register parameters in the hundreds of thousands. This substantial discrepancy highlights the sparse memory utilization inherent in our model, a characteristic that holds substantial significance. It is particularly advantageous when considering the amalgamation of multiple models to bolster accuracy, especially in scenarios necessitating low complexity and an abbreviated runtime. This streamlined memory usage serves as a pivotal advantage, especially in contexts where computational efficiency and rapid processing are imperative.

Moreover, this substantial difference in the parameter count underscores a critical limitation in NN models, notably their impracticality for deployment within SDR systems. The hefty memory requirements demanded by NN architectures, often in the realm of millions of parameters, render them impractical for many SDR implementations. In contrast, our model’s ability to operate within the realm of mere hundreds or thousands of parameters renders it significantly more feasible for integration within SDR systems. This advantage positions our approach as particularly suitable for real-world deployment scenarios, where memory limitations are stringent, allowing for more practical utilization within SDR-based Cognitive Radio networks.

**Figure 4:**
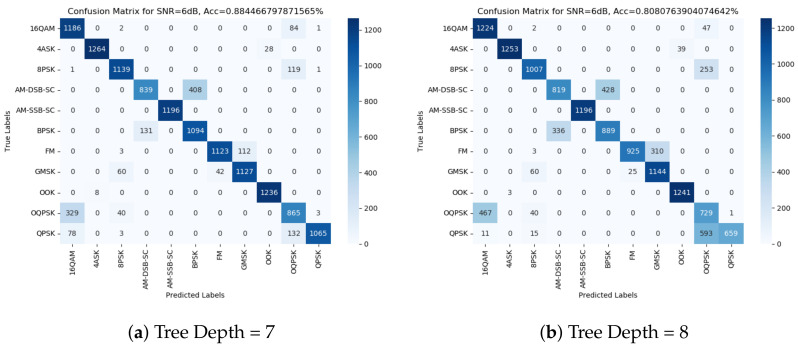
Confusion matrices for different tree depths: (**a**) depth = 7; (**b**) depth = 8.

We examine the confusion matrix from the suggested method. Figure 4 shows the confusion matrices at 6 dB for the 7- and 8-deep decision trees under the 5 dB SNR threshold train set. We observe in the illustrated figures, as well as in the other matrices we examined, that the mislabeling of the model is consistent, meaning the labels that can become mismatched are almost always the same. Based on this observation, we can split the modulation schemes into subgroups as follows:

- Group 0—AM-SSB;
- Group 1—AM-DSB and BPSK;
- Group 2—4ASK and OOK;
- Group 3—FM and GMSK;
- Group 4—8PSK, 16QAM, QPSK and OQPSK.

Group 0 exclusively comprises SSB modulation due to its nearly instantaneous classification across an array of features, even at relatively low SNR values. This consistent and rapid detection is observed consistently across various models tested, establishing SSB as a distinct and easily recognizable modulation type.

On the other hand, Groups 1, 2, and 3 collectively represent pairs of modulations that exhibit a tendency to be confused or mixed up, particularly prevalent at lower SNR levels. This occurrence suggests a noteworthy statistical similarity in the signal models of these modulation schemes. This statistical resemblance often results in misclassification or overlapping characteristics, leading to the assumption that the underlying signal models within these groups share comparable statistical behaviors, thereby complicating their differentiation, especially under lower SNR conditions.

Group 4, encompassing the remaining modulations, portrays a more intricate scenario, where these modulations commonly intermingle without any particular pair exhibiting a significantly higher occurrence than the others. This lack of distinct differentiation signifies a higher level of complexity within this group, where multiple modulation schemes exhibit overlapping statistical features or behaviors, making their individual identification considerably more challenging, especially in contexts where their distinguishing characteristics are blurred due to similarities.

This categorization provides valuable insights into the behavior and detectability of various modulation groups, shedding light on the distinct characteristics observed across different SNR levels and establishing patterns of similarity or complexity among them.

## 4. Discussion

We conducted simulations to validate our hypothesis on the benefits of using a classic feature-based algorithm for AMC. Our initial focus was to examine the impact of individual features on detection accuracy, which led us to remove unnecessary or redundant features during the model’s training phase. We then partitioned the data into high- and low-SNR groups to demonstrate how specific modulation schemes become detectable only above a certain SNR threshold. This finding allowed us to refine our model by focusing on the essential SNR levels relevant to real-world systems. The final phase of our simulation aimed to fine-tune the tree’s hyperparameters to ensure the highest accuracy while maintaining the optimal complexity.

Next, we compared our finalized model against three NN models, which yielded varied results. On one hand, our proposed method exhibited superiority over NN models in terms of detection accuracy, especially beyond the specified threshold SNR values. On the other hand, our method struggled to detect signals with low SNR, where NN models excelled. However, as previously noted, many modulation schemes become irrelevant for demodulation below certain SNR thresholds. Apart from the detection accuracy, we also compared our method with NN models in terms of memory storage. While basic NN models required millions of deep parameters, our straightforward classifier required a maximum of a few hundred parameters, depending on the chosen model. This advantage significantly impacted the end units, as the variance in total parameters directly influenced memory usage and runtime during the classification process. These factors directly contribute to improved battery life, reduced costs, and enhanced speed in desired end units.

However, our suggested method does have limitations. Primarily, the algorithm may not be optimal for discussions involving low-SNR scenarios and modulations that are decodable even with an SNR lower than 2–4 dB. Additionally, we observed instances where certain modulation schemes were erroneously identified even at higher SNR. This occurrence might be attributed to similar carriers and statistics among some modulations, indicating that our method might not be suitable for those specific schemes. Despite these limitations, all outcomes affirm that the classic FB classifier for AMC can be both successful and advantageous when considering its application in real-life Cognitive Radio networks.

## 5. Conclusions

Our research focused on developing a simple yet effective method for automatic modulation classification in spectrum sensing. We found that our proposed method outperforms some of the most widely used neural networks in terms of both accuracy and memory efficiency. To achieve this, we chose the decision tree classifier for its simplicity, which allowed us to identify efficient statistical features like High-Order Cumulants (HOCs) that are specifically tailored for each modulation scheme. Our study went beyond the usual emphasis on digital modulations and explored the possibility of using cumulants to classify analog modulations, which has not been extensively researched before. By identifying specific cumulants for each modulation type, we discovered that there are subsets of modulations that share similar statistical behaviors. This discovery not only helped us understand modulation characteristics better but also provided a foundation for improving classification methodologies. Our approach is optimal for end units within Cognitive Radio (CR) networks, as it achieves high accuracy with minimal parameters, which is crucial for memory and speed efficiency. In our future work, we plan to leverage these identified modulation subgroups to develop a more intricate method that further improves accuracy. We also aim to expand our approach to include a wider range of modulations, making our classification framework more versatile and applicable.

## Figures and Tables

**Table 1:** The best two features for each modulation at SNR = 10 dB.

Modulation	1st Feature	$P_{d}$	2nd Feature	$P_{d}$
SSB	$C_{4.1}$	100%	$C_{4.0}$	100%
DSB	$C_{4.1}^{d}$	74.69%	$C_{4.0}^{d}$	73.34%
BPSK	$C_{2.0}$	99.57%	$C_{4.0}$	92.73%
FM	$C_{4.1}$	99.63%	$C_{2.0}$	99.27%
GMSK	$C_{4.2}$	96.09%	$C_{8.0}$	81.23%
OOK	$C_{2.0}$	95.82%	$C_{2.1}$	93.61%
4ASK	$C_{2.1}$	95.84%	$C_{2.0}$	93.71%
QPSK	$C_{4.0}^{d}$	87.95%	$C_{8.0}^{d}$	86.69%
OQPSK	$C_{4.2}$	76.39%	$C_{4.0}$	60.53%
16QAM	$C_{6.0}^{d}$	100%	$C_{6.3}^{d}$	99.74%
8PSK	$C_{4.0}^{d}$	100%	$C_{4.2}^{d}$	99.87%

**Table 2:** Parameter size of different models.

Model	Total Parameters
6 Deep Tree	63
7 Deep Tree	127
8 Deep Tree	255
ResNet	214,922
LSTM	770,378
DenseNet	542,850
VGG	1,621,780
FLANs	954,747

## Data Availability

This research is based on the published dataset by Timothy James O’Shea, Tamoghna Roy and T. Charles Clancy. Available at https://www.deepsig.ai/datasets (accessed on 26 May 2023).

## Abbreviations

The following abbreviations are used in this manuscript:

AMC	Automatic modulation classification
CR	Cognitive radio
SDR	Software-defined radio
SNR	Signal-to-noise ratio
FB	Feature based
HOS	High-order statistical moments
HOC	High-order cumulants
AWGN	Additive white Gaussian noise
